# Low Social Support among the Elderly

**Published:** 2019-09

**Authors:** Maryam AZARNIVAND, Fahimeh ALIZADEH, Zeinab SOLTANI, Hadi HOJATI, Ali DADGARI, Mohammad Hassan EMAMIAN

**Affiliations:** 1. Student Research Committee, School of Nursing and Midwifery, Shahroud University of Medical Sciences, Shahroud, Iran; 2. School of Nursing and Midwifery, Shahroud University of Medical Sciences, Shahroud, Iran; 3. Center for Health Related Social and Behavioral Sciences Research, Shahroud University of Medical Sciences, Shahroud, Iran

## Dear Editor-in-Chief

The global population of the elderly in 2015 was estimated to be 900 million people, expected to double by 2050 (1). The population of people aged 60 and over in Iran has been predicted to grow to 10 million in 2019 (2).

Social support has been classified into formal (provided by institutions) and informal (provided by family members, neighbors and friends) (3). Family is considered a special source of support for the elderly (4). Despite the importance of social support in the vulnerable elderly community, social support decreases with age (5).

In this cross-sectional research, we investigated people over 60 yr of age living in Shahroud, northeast Iran, during 2016. Overall, 496 male and female elderly were selected randomly, Lubben Social Network Scale (LSNS) (6) was used to assess social support in family, friends, confidential relations, assisting others and life management domains. The questionnaire contained 10 five-option items each scored on a scale from 0 to 5. The final score ranged from 0 to 50. A higher score implied greater social support. The validity and reliability of the Persian version of this questionnaire were verified (unpublished paper).

This study was supported by Shahroud University of Medical Sciences (project number: 9494) and approved by Ethics Committee of this university with the ethical code IR.SHMU.REC.2015.175. Informed consent was taken from all participants.

The total score of social support for the elderly ranged between 0 and 23 with a mean of 9.0 (95% CI: 8.2 – 9.8). This indicated this community was poorly covered by social support. Nearly all participants had very poor social support. This finding is inconsistent with the other results obtained (7, 8) that moderate social support for elderly was about 70%. The reason for such discrepancy in results seems to be due to different tools employed to measure social support. The tool used in this study was exclusive for the elderly, whereas tools used in other studies focused on almost all ages, i.e. not designed to measure social support for the elderly in particular.

Elderly people in Shahroud City benefited from very poor social support. Since the number of elderly people is rising, it is critical to address their needs as a vulnerable community in the large-scale social, economic and cultural plans.
